# Flavonoid Glycosides and Phenolic Acids from Inula Oculus-Christi Modulate Membrane Organization and Provide Antioxidant Protection

**DOI:** 10.3390/molecules30132740

**Published:** 2025-06-25

**Authors:** Ralitsa Veleva, Tanya Topouzova-Hristova, Aneliya Kostadinova, Dayana Benkova, Antoaneta Trendafilova, Viktoria Ivanova, Veselina Moskova-Doumanova, Kirilka Mladenova, Jordan Doumanov, Vesela Yordanova, Galya Staneva

**Affiliations:** 1 Faculty of Biology, Sofia University ‘St. Kliment Ohridski’, 1164 Sofia, Bulgaria; ralitsa_veleva@biofac.uni-sofia.bg (R.V.); moskova@biofac.uni-sofia.bg (V.M.-D.); k_mladenova@biofac.uni-sofia.bg (K.M.); doumanov@biofac.uni-sofia.bg (J.D.); 2 Centre of Competence “Sustainable Utilization of Bio-Resources and Waste of Medicinal and Aromatic Plants for Innovative Bioactive Products” (BIORESOURCES BG), 1000 Sofia, Bulgaria; antoaneta.trendafilova@orgchm.bas.bg (A.T.); viktoria.genova@orgchm.bas.bg (V.I.); 3 Institute of Biophysics and Biomedical Engineering, Bulgarian Academy of Sciences, Acad. G. Bonchev Street, 1113 Sofia, Bulgaria; aneliakk@yahoo.com (A.K.); dayanabenkova369@abv.bg (D.B.); vyordanova@biophys.bas.bg (V.Y.); 4 Institute of Organic Chemistry with Centre of Phytochemistry, Bulgarian Academy of Sciences, 1113 Sofia, Bulgaria

**Keywords:** flavonoid glycosides, phenolic acids, Inula oculus-christi, lipid order, rafts, A549 cells

## Abstract

Oxidative stress induces lipid peroxidation within the membrane bilayer, thereby compromising membrane integrity. Polyphenols (PPs), renowned for their antioxidant properties, have been shown to mitigate oxidative damage. Here, we investigated the structural and antioxidant effects of PPs—specifically flavonoid glycosides (FGs) and phenolic acids (PAs)—extracted from *Inula oculus-christi* using steady-state fluorescence spectroscopy in both model and cell membranes. Membrane lipid order was evaluated using DPH and Laurdan spectroscopy, while DPH-TEMPO fluorescence quenching was employed to quantify raft-like domain formation in model systems. The antioxidant capacity of the PP extracts was assessed via fluorescence quenching of *cis*-parinaric acid. Both FGs and PAs conferred approximately 2-fold antioxidant protection, with FGs showing a 1.13-fold greater effect than PAs. In addition, both PP classes promoted lipid raft formation, particularly in cholesterol-rich membranes. PPs increased order in the liquid-disordered (L_d_) phase while inducing disorder in the liquid-ordered (L_o_) phase, depending on the lipid-to-PP ratio. Notably, FGs enhanced membrane fluidity more strongly in A549 than in MDCKII cells, as reflected by a ~5.7-fold decrease in Laurdan GP in A549 (from 0.04 to −0.17) versus a ~1.4-fold decrease in MDCKII at 200 μg/mL. These findings highlight the dual structural and antioxidative roles of FGs and PAs in preserving membrane integrity under oxidative stress.

## 1. Introduction

Plants of the genus *Inula*, native to the Mediterranean regions of Europe, Asia, and Africa, have been extensively used in folk remedies for asthma, coughs, excessive phlegm, arthritis, and back pain. Numerous studies have documented the diverse therapeutic properties of *Inula* species, including anti-inflammatory, antiviral, antifungal, cardioprotective, antianginal, and antidiabetic properties [1,2,3].

One of the predominant classes of biologically active plant compounds in *Inula* species is polyphenols (PPs), particularly flavonoids and phenolic acids. Flavonoids are hydroxylated phenolic substances based upon a fifteen-carbon skeleton (C_6_-C_3_-C_6_) and can occur as aglycones, glycosides, and methylated derivatives. Among them, flavonoid C- and O-glycosides (FGs) exhibit significant bioactivity [4,5]. Phenolic acids (PAs) are hydroxylated compounds with a carboxylic group, classified into hydroxybenzoic (C_6_-C_1_) and hydroxycinnamic (C_6_-C_3_) acids based on their carbon structure. They typically occur in bound forms (amides, esters, or glycosides) rather than as free acids [6,7].

FGs and PAs have a broad and extensively documented range of biological activities, including immunomodulatory and anti-inflammatory [8,9,10], anticancer [11,12,13], antimicrobial [14,15], and antidiabetic [16] effects. The protective and health-promoting activities of these PPs are largely attributed to their potent antioxidant properties, which have been widely documented through various assays. Both FGs [17,18] and PAs [19,20] have demonstrated remarkable antioxidant efficacy, surpassing even conventional antioxidants such as vitamins C, E, and β-carotene [21]. Due to their antioxidant potencies, FGs and PAs from *Inula* species hold immense potential in pharmacological applications, particularly in the prevention of diseases associated with cellular oxidative stress. One of the primary cellular targets of oxidative damage is the lipid membrane, where peroxidation disrupts key properties such as fluidity, permeability, and structural integrity [22,23]. Studies have shown that FGs and PAs mitigate membrane oxidative damage by scavenging peroxyl radicals [24,25], reducing lipid hydroperoxides [26,27], and suppressing secondary aldehyde formation [27,28].

Despite numerous studies on phenolic compounds, a comprehensive analysis of the effects of FGs and PAs on cell membrane architecture remains lacking. Such studies are essential, as they can help elucidate the mechanisms underlying the protective roles of these compounds against oxidative membrane damage. Moreover, the literature lacks studies that employ model systems accurately replicating the native lipid composition of cell membranes, which are essential for deciphering the specific interactions of polyphenols with different lipid classes and lipid phases. The eukaryotic cell membrane exhibits a specific lateral phase heterogeneity. Cholesterol (Chol) and saturated phospholipids form tightly packed, liquid-ordered (L_o_) phases, which coexist with surrounding liquid-disordered (L_d_) phases enriched in unsaturated phosphoglycerolipids [29,30,31]. The phase segregation contributes to the formation of specialized L_o_ nanodomains, known as lipid rafts [32], which play a crucial role in cell signaling, membrane trafficking, interactions with the cell cytoskeleton, cell migration, and cell adhesion [33,34,35,36,37].

This study aims to investigate the interactions of FGs and PAs derived from Bulgarian *Inula oculus-christi* with model membranes using a suite of spectroscopic techniques. These include Laurdan fluorescence spectroscopy, DPH polarization, DPH-TEMPO quenching, and *cis*-parinaric acid fluorescence assays. Model membranes are composed of lipid mixtures that accurately model the lipid phase heterogeneity of the plasma cell membranes. Key membrane properties—such as lipid order, fluidity, polarity, and raft domain dynamics—are quantitatively assessed following treatment with the polyphenols. Additionally, the study is extended to living cells to examine how these compounds influence membrane organization, thereby offering a more comprehensive understanding of their antioxidant mechanisms and membrane-targeted bioactivity.

## 2. Results

### 2.1. Effects of Plant Metabolites on Membrane Models

#### 2.1.1. Effects of PPs on the Phase Transition of EggSM

Sphingomyelin (SM) is a prevalent lipid in eukaryotic membranes, characterized by long and saturated fatty acid chains. This lipid plays a pivotal role in membrane organization phase behavior due to its tight molecular packing and asymmetric molecular structure [38,39,40,41]. Notably, SM exhibits a phase transition with a main transition temperature (T_m_) that falls within the physiological temperature range (37–41 °C) [42,43]. SM, the most abundant sphingolipid in the membrane, influences membrane fluidity and plays a key role in the formation of nanodomains. To assess the impact of FGs and PAs on the phase transition of SM-containing membranes, DPH fluorescence anisotropy measurements were performed using large unilamellar vesicles (LUVs) composed of EggSM. The experiments were conducted over a broad temperature range (20–51 °C) with different EggSM/PPs ratios (250/1, 100/1 and 50/1). The rationale for selecting the EggSM/PPs ratios (250/1, 100/1, and 50/1) is provided in the Section 4. The effects were evaluated by measuring alterations in membrane fluidity at the acyl chain region.

**Figure 1:**
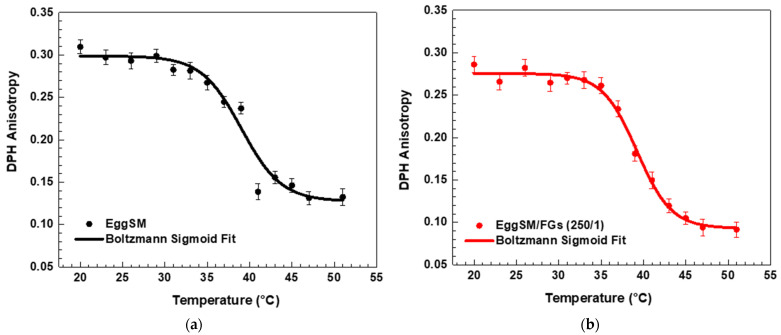

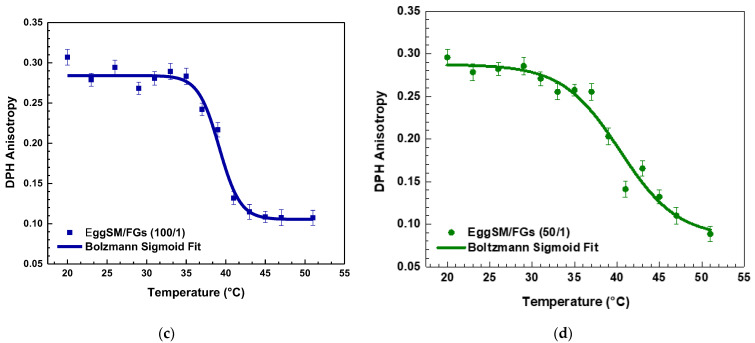
DPH anisotropy of EggSM phase transition—effects of FGs. Measurements were taken at 3 °C increments from 20 to 29 °C and at 2 °C increments from 29 to 51 °C. Results are presented as means ± standard deviations (SD) of three independent measurements per sample (*n* = 3). The solid line represents a sigmoid curve fit applied to the experimental data. Phase transition of control EggSM LUVs (**a**); phase transition of FG-containing LUVs in different EggSM/FGs ratios: (**b**) 250/1, (**c**) 100/1, and (**d**) 50/1.

**Figure 2:**
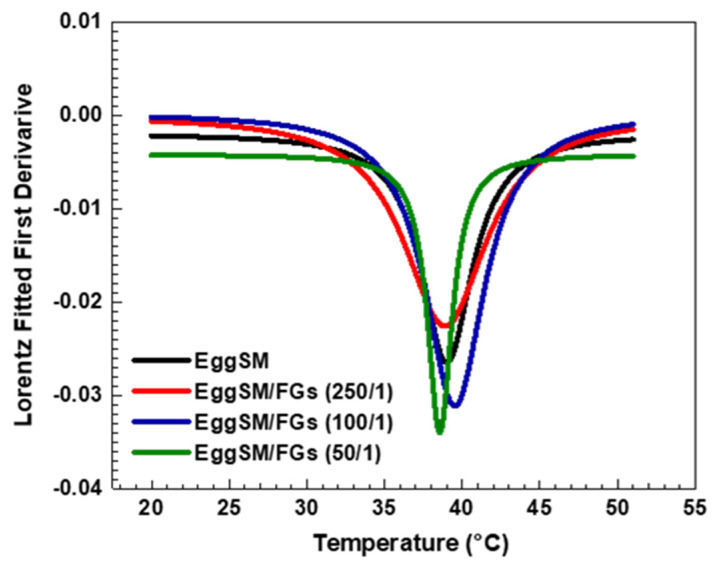
The first derivative of DPH anisotropy during the EggSM phase transition at different FGs ratios fitted by Lorentz function (fitting parameters in SI).

A characteristic sigmoidal curve of EggSM phase transition was observed, representing the transition from the gel phase (L_β_ or S_o_) to the liquid-disordered phase (L_d_ or L_α_) (Figure 1a). A detailed information about the sigmoid fit is given in Supplementary Information (SI). The incorporation of FGs into EggSM LUVs resulted in a shift of the phase transition sigmoidal curve toward lower anisotropy (*r*) values (Figure 1b–d), suggesting an increase in membrane fluidity during the phase transition. Further analysis, using the first derivative of the sigmoidal curve fitted to a Lorentzian peak function (Figure 2, fitting parameters in SI) confirmed that the T_m_ of EggSM is 39.3 ± 0.6 °C (Table 1). A shift in the EggSM T_m_ to 38.2 ± 0.4 °C was observed at the highest EggSM/FGs ratio of 50/1 (Figure 2) (Table 1).

**Figure 3:**
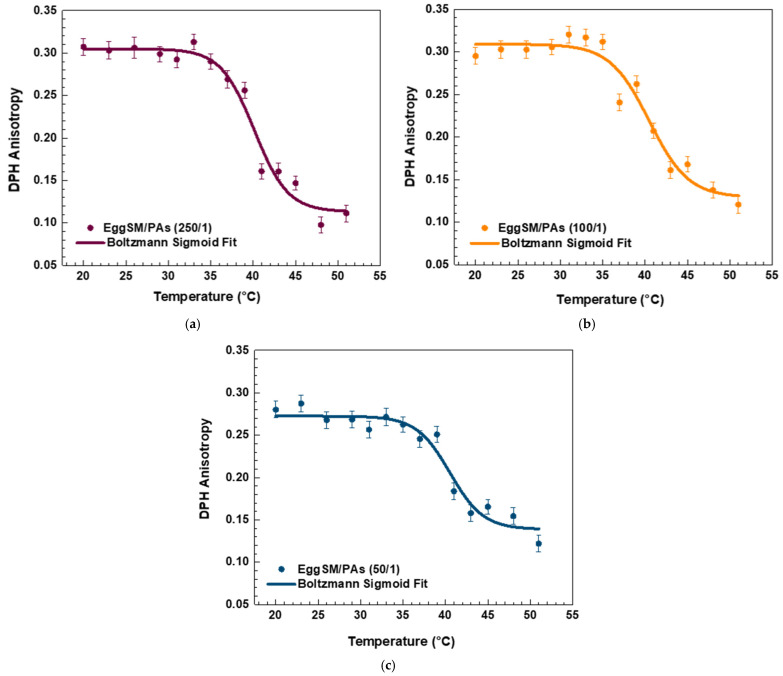
DPH anisotropy of EggSM phase transition—effects of PAs. Measurements were taken at 3 °C increments from 20 to 29 °C and at 2 °C increments from 29 to 51 °C. Results are presented as means ± standard deviations (SD) of three independent measurements per sample (*n* = 3). The solid line represents a sigmoid curve fit applied to the experimental data. Phase transition of PA-containing LUVs in different EggSM/PAs ratios: (**a**) 250/1, (**b**) 100/1, and (**c**) 50/1.

**Figure 4:**
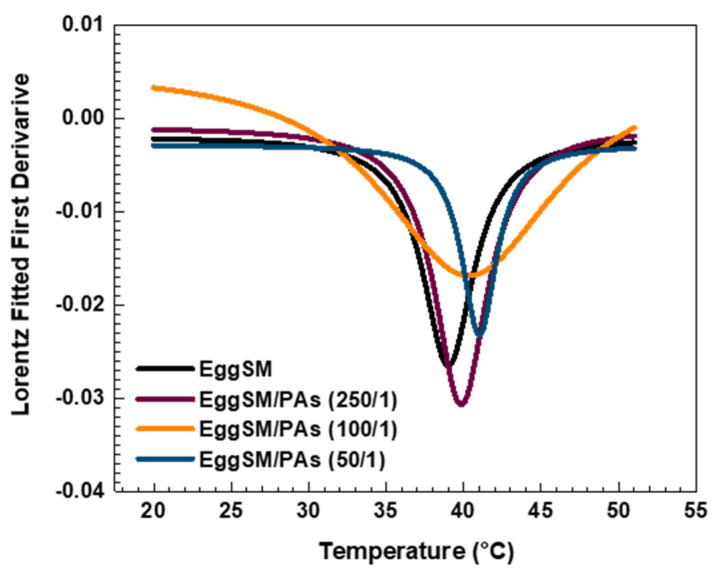
The first derivative of DPH anisotropy during the EggSM phase transition at different PAs ratios fitted by Lorentz function (fitting parameters in SI).

The effect of PAs on the phase transition of EggSM LUVs was evaluated using the same experimental protocol. The presence of PAs induced a shift in the sigmoidal curve of the EggSM phase transition toward higher *r* values, indicating a reduction in membrane fluidity during the phase transition (Figure 3a–c). Moreover, at higher EggSM/PAs ratios of 100/1 and 50/1 (Figure 4), the T_m_ of EggSM increased from 39.3 ± 0.6 °C to 41.3 ± 0.7 °C (Table 1), suggesting a stabilizing effect of PAs on the membrane structure. Strikingly, the two polyphenols exhibit slight effects but opposing ones on the phase transition of EggSM membranes: FGs tend to decrease the transition temperature, whereas PAs tend to increase it. The Lorentzian peak width (LPW) gives information about the phase transition’s temperature range and degree of phase heterogeneity. The most pronounced increase in LPW was observed at an EggSM/FGs ratio of 250/1 and an EggSM/PAs ratio of 100/1 (Figure 2 and Figure 4) (Table 1). This expansion of the phase transition temperature range suggests an extended coexistence of the L_β_ and L_d_ phases, leading to an increased heterogeneity in the system. However, at the highest tested ratio of 50/1 for both EggSM/FGs and EggSM/PAs (Figure 2 and Figure 4) (Table 1), a significant reduction in LPW was detected. This indicates a narrowing of the phase transition temperature range, likely due to the facilitated acyl chain melting of EggSM, thereby increasing the phase homogeneity within the system.

#### 2.1.2. Effects of FGs and PAs on the Lipid Order of the L_o_ Raft-like Phase, Composed of EggSM/Chol

The binary lipid mixture of EggSM/Chol (1/1) served as a model for the raft-like L_o_ phase. The impact of varying concentrations of FGs and PAs was assessed using DPH fluorescence anisotropy and Laurdan fluorescence spectroscopy. These two fluorescent probes, positioned at different depths within the lipid bilayer, provided complementary insights into the effects of FGs and PAs on membrane order and fluidity.

DPH fluorescence anisotropy: The incorporation of cholesterol (Chol) into the SM-containing membranes decreases the membrane fluidity. When Chol is present at concentrations exceeding 33 mol% in model membranes, EggSM membranes are in L_o_ phase [44].

**Figure 5:**
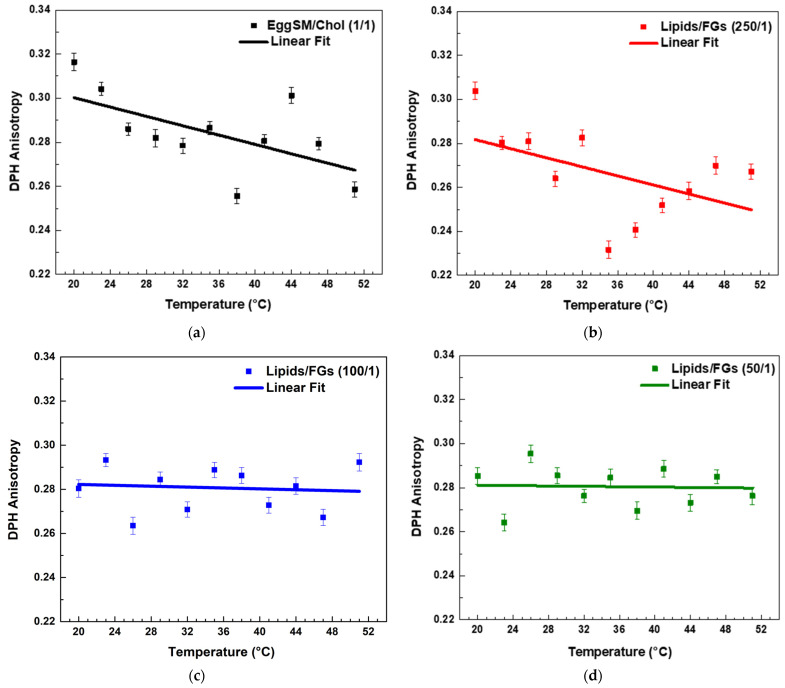
DPH anisotropy of raft-like L_o_ phase measured across a temperature range of 20–51 °C—effects of FGs. Results are presented as means ± standard deviations (SD) of three independent measurements per sample (*n* = 3). Solid line represents a linear regression analysis (fitting parameters in SI). DPH anisotropy of control EggSM/Chol (1/1) LUVs (**a**); DPH anisotropy of FG-containing LUVs in different lipids/FGs ratios: (**b**) 250/1, (**c**) 100/1, and (**d**) 50/1.

Both PPs decreased the *r* values in the raft-like L_o_ phase, indicating an increase in the fluidity around the acyl chains. A linear regression analysis was performed to examine the temperature dependence of DPH anisotropy in EggSM/Chol (1:1 molar ratio) membranes with and without PPs (see SI for more details in fitting parameters). The data showed a linear decrease in anisotropy with increasing temperature. The linear fit (solid line) (Figure 5a) indicates a negative correlation, suggesting a progressive increase in membrane fluidity with rising temperature. In the sample with a lipids/FGs ratio of 250/1 (Figure 5b), a similar negative linear trend was observed, indicating that DPH anisotropy decreased progressively with increasing temperature from 20 °C to 50 °C. This decrease in anisotropy reflects enhanced membrane fluidity at higher temperatures and suggests that at this low FG concentration, the bilayer maintains its typical temperature sensitivity, similar to the untreated control system. In contrast, at a lipids/FGs ratio of 100/1 (Figure 5c), the linear fit of the anisotropy data was nearly horizontal, and the fluctuations in anisotropy across the temperature range were minimal. These findings indicate that membrane fluidity remained relatively stable, suggesting that FGs at this intermediate concentration can buffer or attenuate the temperature-induced changes in membrane dynamics. A similar trend was observed at the lipids/FGs ratio of 50/1 (Figure 5d), where the anisotropy values remained largely constant with increasing temperature. The absence of a significant temperature dependence implies that higher FG concentrations contribute to a tight membrane packing of the lipid bilayer, effectively dampening the thermally driven increase in membrane fluidity.

**Figure 6:**
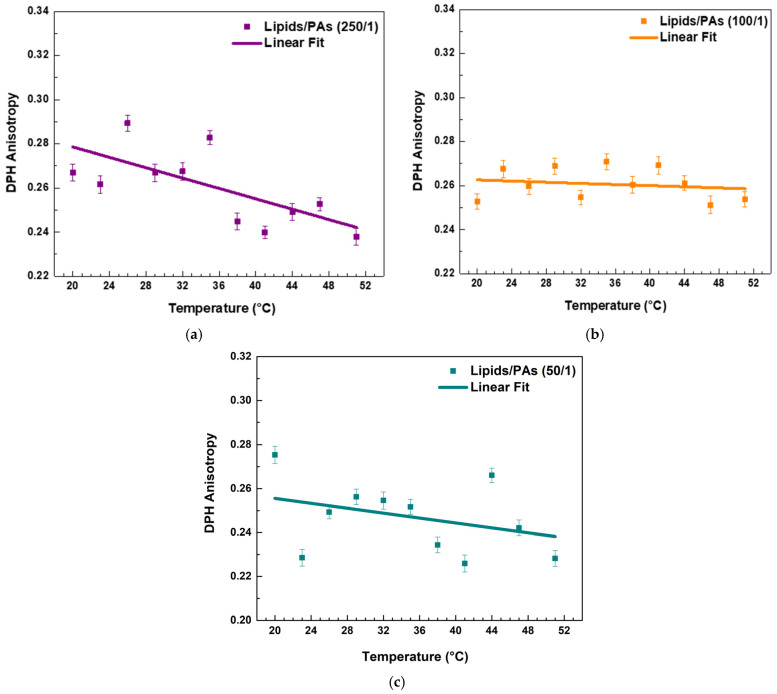
DPH anisotropy of raft-like L_o_ phase measured across a temperature range of 20–51 °C—effects of PAs. Results are presented as means ± standard deviations (SD) of three independent measurements per sample (*n* = 3). Solid line represents a linear regression analysis (fitting parameters in SI). DPH anisotropy of PA-containing LUVs in different lipids/PAs ratios: (**a**) 250/1, (**b**) 100/1, and (**c**) 50/1.

**Figure 7:**
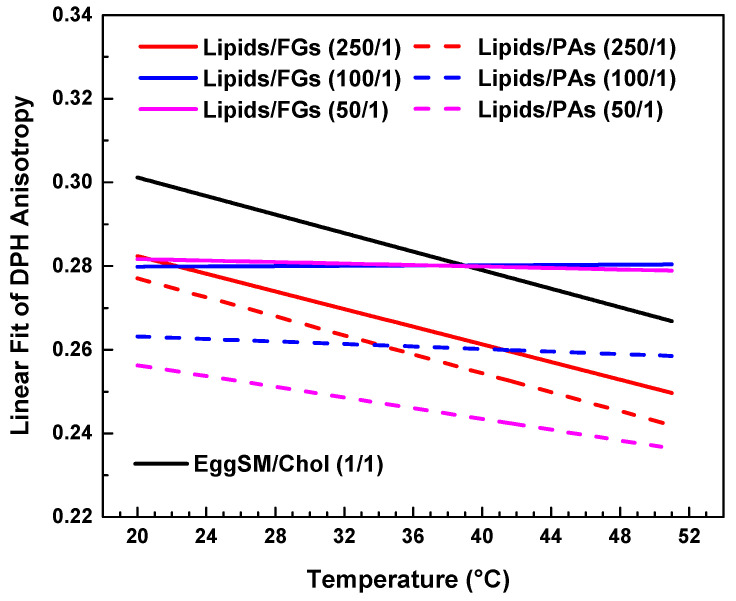
Linear fits of DPH anisotropy of raft-like L_o_ phase with FGs (solid lines) and PAs (dashed lines). Data is collected in a single graph only for a clearer comparison between the effects of all studied lipids/PPs ratios.

PAs affected the fluidity of the raft-like L_o_ phase. Both the highest and lowest lipids/PAs ratios of 50/1 and 250/1 caused a noticeable decrease in *r* values with temperature, implying a fluidizing effect (Figure 6a,c and Figure 7). However, the 100/1 ratio (Figure 6b) showed a similar effect to FGs (Figure 5c), resulting in a permanent fluidizing effect without any significant transition in the system across the temperature range (Figure 7).

Comparative effect of FGs and PAs on membrane fluidity and thermal responsiveness.

Both types of PPs influenced membrane structure in a concentration-dependent manner, but with distinct trends (Figure 7). When comparing the absolute values of DPH anisotropy, it becomes evident that PAs reduce DPH anisotropy more effectively than FGs, indicating a stronger fluidizing effect with increasing PA concentration.

At the lowest additive concentration (250/1), both FGs and PAs caused a linear decrease in DPH anisotropy with increasing temperature, indicating that membrane fluidity increased with temperature in both systems. However, the slope of the linear fit was slightly steeper in the PA-treated membranes (Figure 7, fitting parameters in SI). This implies that at low concentrations, PAs may exert a less-pronounced temperature-dependent effect on membrane fluidity compared to FGs.

At intermediate concentration (100/1), the FG-containing membranes showed minimal temperature dependence, with an almost flat slope and a slightly negative adjusted R^2^ (fitting parameters in SI), indicating effective suppression of thermal disordering. In contrast, PA-treated membranes at the same ratio still displayed a negative slope, suggesting that PAs are less effective than FGs at buffering thermally induced changes in fatty acids packing at this concentration.

At the highest concentration (50/1), FGs nearly completely abolished temperature-induced changes in anisotropy, with an adjusted R^2^ of −0.11 and an essentially zero slope (SI). PA-treated membranes, however, retained a modest temperature-dependent decrease in anisotropy, with a small but measurable slope.

In summary, while both FGs and PAs modulate membrane fluidity in a concentration-dependent manner, FGs exhibit a greater ability to suppress the thermal dependence of membrane fluidity, particularly at intermediate and high concentrations. This suggests that FGs may integrate more deeply into the bilayer and induce stronger fatty acid packing effects compared to PAs.

**Figure 8:**
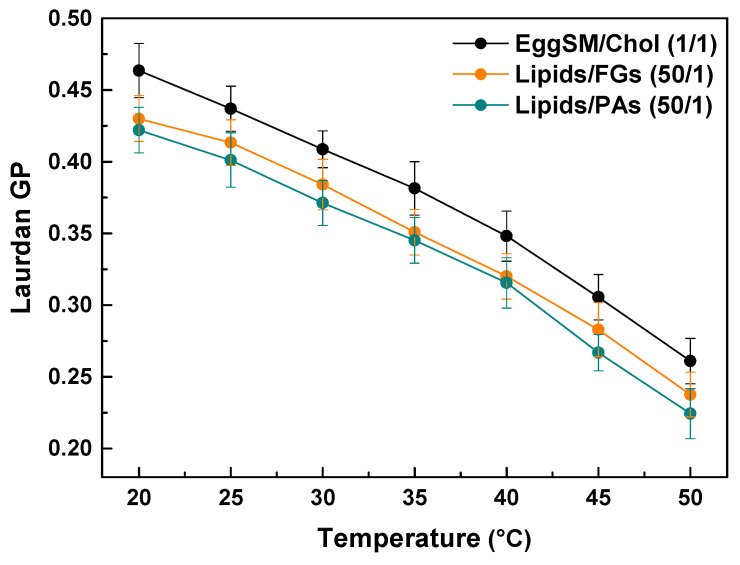
Laurdan GP values of raft-like L_o_ phase, composed of EggSM/Chol (1/1) as a function of temperature. Effects of FGs and PAs at a lipids/PPs ratio of 50/1. Measurements were made across a temperature range of 20–50 °C with a 5 °C increase per measurement. Results are presented as means ± standard deviations (SD) of three independent measurements per sample (*n* = 3) at each temperature.

Laurdan fluorescence spectroscopy was employed to evaluate the effect of PAs and FGs on the lipid order of the raft-like L_o_ phase. The measurements were performed within the temperature range from 20 to 50 °C with a 5 °C increment per measurement. The control exhibits a temperature-dependent decrease in the GP values, indicating a decrease in lipid order at the hydrophilic–hydrophobic membrane interface. Both FGs and PAs caused a temperature-dependent decrease in the GP values similar to the control. Noticeable concentration-dependent shifts were observed towards lower GP values compared to the control (Figure 8). Moreover, PAs exhibited a slightly stronger effect. These findings imply that both FGs and PAs cause a temperature-dependent decrease in the lipid order at the hydrophobic–hydrophilic level of the raft-like L_o_ phase.

#### 2.1.3. Effects of FGs and PAs on the Lipid Order of Highly Fluid L_d_ Phase, Composed of EggPC

**Figure 9:**
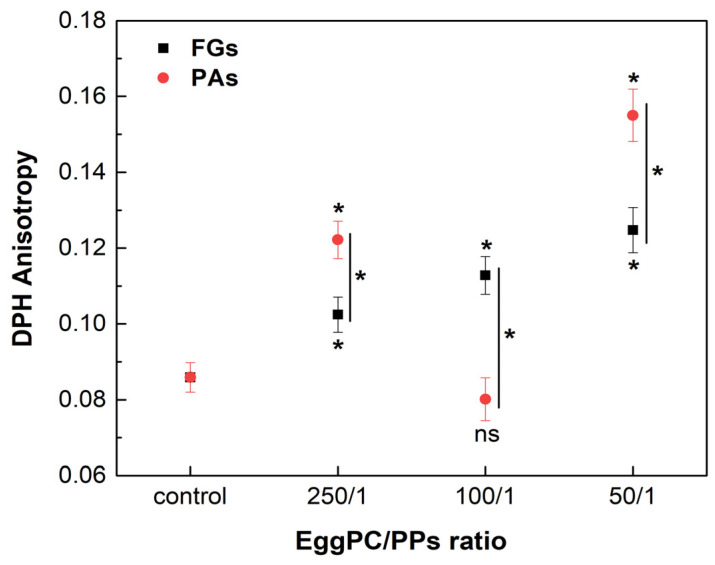
DPH anisotropy of fluid L_d_ phase, composed of EggPC and different lipids/PPs ratios. Measurements were carried out at 37 °C. Results are presented as means ± standard deviations (SD) of three independent measurements per sample (*n* = 3). Statistical significance is denoted by * (0.05), comparing each ratio of lipids/PPs with the control (untreated EggPC LUVs). Statistical significance between the effects of the FGs and PAs at the same lipids/PPs ratio is denoted by |* (0.05). A lack of statistical significance is indicated by ns (not significant).

Phosphatidylcholine (PC) is the most abundant lipid in the eukaryotic membranes, accounting for 40 to 60% [45]. Unsaturated PC species form the highly fluid L_d_ phase in the membrane bilayer at physiological temperature. The effects of FGs and PAs on the fluidity of the L_d_ phase were assessed by DPH anisotropy measurements, using EggPC as a lipid model. The measurements were carried out at a temperature of 37 °C. FGs caused a concentration-dependent increase in the *r* values in the highly fluid L_d_ phase, suggesting an increase in the membrane order at the acyl chains of the lipid (Figure 9). On the other hand, the lowest and the highest concentrations of PAs induced a significant increase in the *r* values observed, exceeding the control and the ordering effect of FGs. However, at a lipids/PAs ratio of 100/1, a decrease in the *r* value occurred, suggesting a fluidizing effect.

#### 2.1.4. Effect of FGs and PAs on the Relative Size of Raft-like L_o_ Domain Fraction in Ternary Mixtures Composed of EggPC/EggSM/Chol

Ternary lipid mixtures composed of EggPC, EggSM, and Chol were used as a model system to mimic the phase heterogeneity of biological membranes. At physiological temperature, phase coexistence is expected, with raft-like L_o_ domains surrounded by a more fluid liquid-disordered L_d_ phase. The effect of both PPs on the lipid raft-like fraction was assessed using quenching of DPH fluorescence by TEMPO. To ensure physiological relevance, three different lipid ratios were selected, resembling those found in native cell membranes. Raft-like L_o_ domains typically consist of SM and Chol in a 1/1 to 2/1 ratio. Based on this, the following lipid mixtures were used: EggPC/EggSM/Chol at 40/40/20 (representing a common plasma membrane composition), 50/25/25, and 33/33/34 (where Chol is equimolar to EggSM).

**Figure 10:**
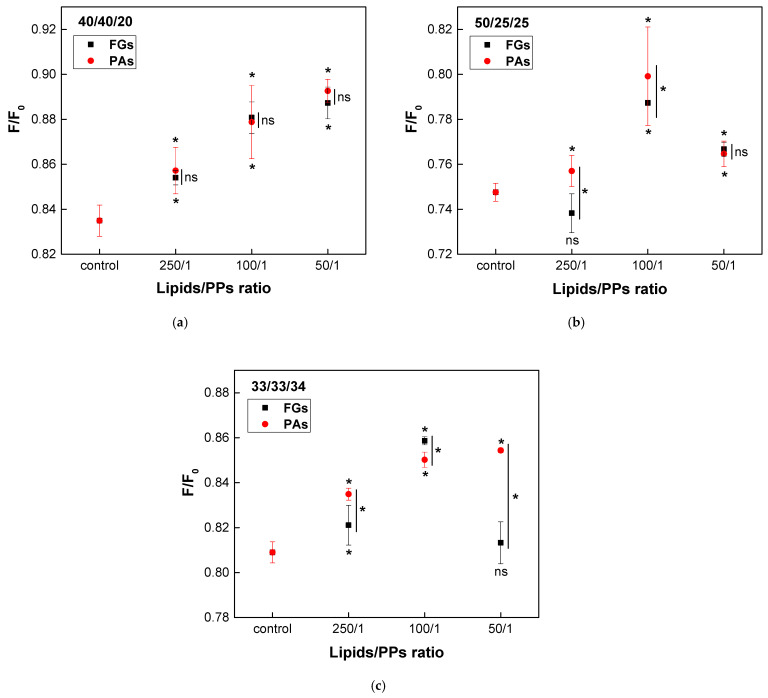
Effects of FGs and PAs on DPH fluorescence quenched by TEMPO. F/F_0_ as a function of lipids/PPs ratios in three types of heterogenous bilayers, composed of EggPC/EggSM/Chol: (**a**) 40/40/20; (**b**) 50/25/25; (**c**) 33/33/34 (mol/mol). Measurements were carried out at 37 °C. Results are presented as means ± standard deviations (SD) of three independent measurements per sample (*n* = 3). Statistical significance is denoted by * (0.05), comparing each ratio of lipids/PPs with the respective control (untreated EggPC/EggSM/Chol LUVs). Statistical significance between the effects of FGs and PAs at the same lipids/PPs ratio is denoted by |* (0.05). A lack of statistical significance is indicated by ns and |^ns^.

A concentration-dependent increase in the raft fraction across all three types of heterogeneous bilayers was induced by both PPs (Figure 10). In the model system with the lowest Chol concentration (40/40/20), both FGs and PAs exhibited a similar concentration-dependent enhancement of raft formation, with no significant differences in their effects (Figure 10a). In vesicles composed of the second ternary mixture (50/25/25), the maximum increase in the raft fraction was observed at a lipids/PPs ratio of 100/1 for both FGs and PAs (Figure 10b). However, a reduction in the raft fraction was detected only at the lowest concentration of FGs (250/1). The most significant difference in the effects of FGs and PAs was observed in membranes with a lipid ratio of 33/33/34, which contained the highest Chol content. The activity of PAs exhibited a direct concentration-dependent effect, whereas FGs reached their maximum impact at a lipids/FGs ratio of 100/1 (Figure 10c).

### 2.2. Antioxidant Properties of Polyphenols, Tested on Model Membranes of Unsaturated Lipids

**Figure 11:**
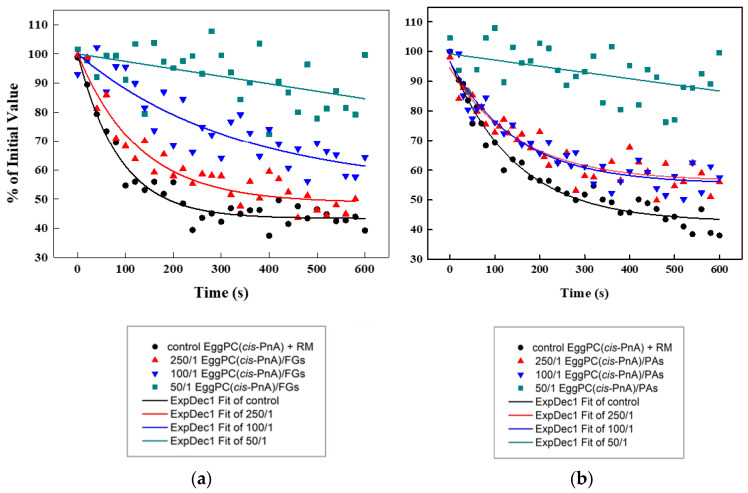
Fluorescence quenching of *cis*-PnA in control EggPC LUVs and PP-containing ones as a function of time: (**a**) effects of FGs; (**b**) effects of PAs.

The antioxidant capacities of FGs (Figure 11a) and PAs (Figure 11b) were evaluated using a *cis*-parinaric acid (*cis*-PnA) fluorescence assay and model membranes composed of unsaturated EggPC. Due to their high susceptibility to free radical-induced oxidation, unsaturated lipids serve as an ideal model for assessing the antioxidant potential of both PPs. The *cis*-PnA possesses multiple conjugated double bonds, and its intrinsic fluorescence makes the acid highly sensitive to oxidation, enabling precise detection via spectroscopy measurements.

To assess the antioxidant properties of both PPs, the fluorescence quenching of parinaric acid was measured following exposure to an oxidizing reaction mixture (RM) (200 μM CuSO_4_, 3 μM H_2_O_2_, 200 μM EDTA). The control EggPC LUVs exhibited the highest level of oxidation (Figure 11). The presence of PPs provided a significant protective effect, reducing the percentage of oxidized lipids nearly 2-fold compared to controls. At a lipids/PPs ratio of 50:1, the results showed a ~2.0-fold antioxidant effect for FGs and ~1.78-fold for PAs relative to the control, with FGs demonstrating ~1.13-fold greater efficacy than PAs.

**Figure 12:**
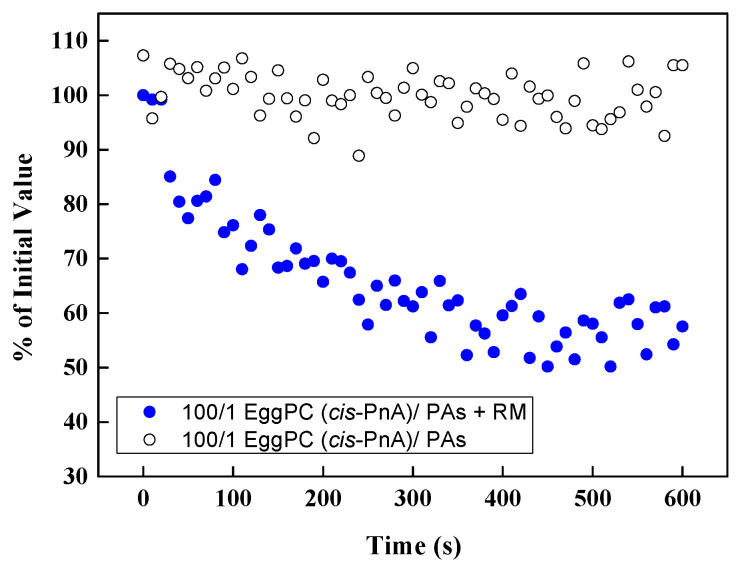
Effect of PAs in EggPC LUVs on the fluorescence of *cis*-PnA with and without the reaction mixture (RM) as a function of time.

To confirm that the fluorescence of *cis*-parinaric acid was not directly influenced by polyphenols, we conducted control measurements in the absence of an oxidizing reaction mixture (Figure 12). No significant variations in fluorescence signal were observed in the presence of PPs, indicating that the measured antioxidant effects were not due to direct interference with fluorescence but rather the protective action of PPs against lipid oxidation.

### 2.3. Effect of FGs and PAs on Cell Membrane Lipid Order

**Figure 13:**
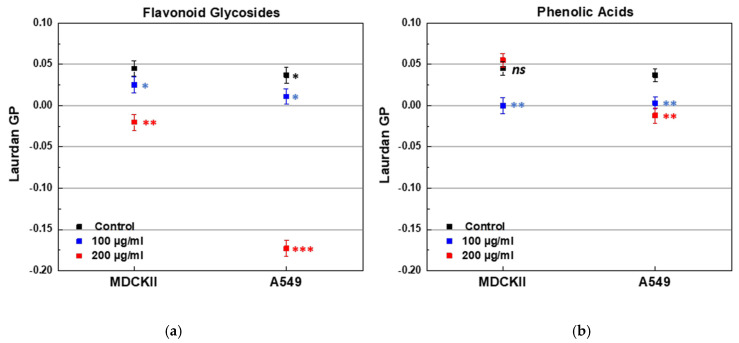
Laurdan GP of MDCKII and A549 cell lines treated with FGs and PAs in two aliquot concentrations of 100 and 200 µg/mL. (**a**) Effect of FGs; (**b**) effect of PAs. * (black) denotes a statistically significant difference (*p* < 0.05) between both cell lines; blue and red stars denote a statistically significant difference (* for *p* < 0.05; ** for *p* < 0.01; *** for *p* < 0.001) between control cell line and PP-containing sample.

The effect of both PPs on the lipid order of cell membranes was evaluated by Laurdan fluorescence spectroscopy. Two types of live, non-fixed epithelial cell lines were used, differing in their origin and malignancy. The first cell line, MDCKII, was a non-cancerous renal epithelial cell line, which can polarize and is expected to exhibit more ordered membranes. The second line, A549, was a cancerous lung epithelial cell line (type 2 pneumocytes) with inherently more fluid membranes. The initial membrane lipid order of the cell lines was confirmed by the control GP values, 0.037 for A549 and 0.045 for MDCK cell lines. Treatment with FGs resulted in a concentration-dependent decrease in membrane order in both cell lines, as indicated by lower GP values (Figure 13a). The most pronounced effect was observed in A549 cells treated with 200 µg/mL of FGs (Figure 13a). PAs induced a concentration-dependent decrease in GP values of A549 membranes (Figure 13b). In MDCKII cells, membrane fluidity increased at 100 µg/mL of PAs, but at 200 µg/mL, a slight decrease in fluidity was observed, suggesting a concentration-dependent biphasic effect (Figure 13b). Overall, FGs induced a markedly greater increase in membrane fluidity in A549 cells compared to MDCKII cells, as evidenced by a ~5.7-fold reduction in Laurdan GP (from 0.034 to −0.173) in A549, versus only a ~1.4-fold decrease in MDCKII following treatment with 200 μg/mL.

These findings highlight a significant difference in how FGs affect cancerous and non-cancerous cells. The pronounced membrane fluidization observed in A549 cells suggests a potential mechanism by which these compounds may compromise cancer cells’ integrity, potentially leading to disruptions in their development and survival.

## 3. Discussion

### 3.1. Interactivity Between PPs and Model Membranes in Different Phase States

The interactions between polyphenols (PPs) and cell membranes are strongly influenced by the chemical structures of the PPs, particularly their lipophilicity and water solubility. These physicochemical properties play a pivotal role in determining how polyphenols modulate cell membrane structure. In the fraction enriched in flavonoid glycosides (FGs), the presence of flavone nepetrin/hispidulin-7-O-glucoside was confirmed. The attachment of a glycosidic moiety at the 7-O position imparts amphiphilic characteristics to the FG, which contributes to their distinct behavior in biological environments [5]. The fraction enriched in phenolic acids (PAs) was found to contain chlorogenic acid (CGA) as well as several dicaffeoylquinic acid (diCQA) isomers, including 1,5-, 3,5-, 4,5-, and 3,4-dicaffeoylquinic acids. These compounds are predominantly hydrophilic, owing to their multiple hydroxyl (-OH) and carboxyl (-COOH) functional groups, which enhance their capacity for hydrogen bonding and thus increase water solubility [46].

The use of complementary spectroscopic techniques enabled a detailed assessment of the effects of FGs and PAs on bilayer structural alterations across different membrane depths, spanning from the polar headgroup region to the hydrophobic core. DPH polarization measurements revealed that both PPs influenced the phase behavior of EggSM. Specifically, both FGs and PAs modulated the phase transition of the bilayer, but in contrasting ways: FGs induced a shift toward a more fluid, disordered state, whereas PAs promoted a more ordered and rigid bilayer structure. Moreover, both FGs and PAs affected the lipid organization within the liquid-ordered (L_o_) raft-like phase by decreasing the lipid packaging at the polar and the hydrophobic regions of the membrane. According to studies, flavonoids interact with membranes and affect their fluidity depending on the lipid composition and the flavonoid concentrations [47]. It has been documented that flavonoids increase the membrane fluidity in tightly packed, rigid membranes by penetrating the hydrophobic bilayer core, acting as a spacer, and disordering the lipids, thereby affecting the lipids’ T_m_ [48]. To date, there is no direct experimental evidence specifically characterizing the interaction of nepetrin with lipid bilayers, particularly with the raft-like L_o_ phase. Nonetheless, insights can be drawn from structurally related compounds. For example, hesperidin, a well-characterized flavanone-7-O-glycoside, has been reported to fluidize cholesterol-rich binary membrane models [49]. Apart from hydrophobic interactions, the amphiphilic nature of FGs promotes interaction with the hydrophilic regions of biological membranes. This property enables FGs to associate with the polar head groups of membrane lipids, primarily through electrostatic interactions [50]. In particular, the polar sugar moiety of hispidulin-7-O-glucoside is likely oriented toward the water medium at the level of the hydrated polar head groups of the membrane lipids. This orientation enhances the polarity at the membrane–water interface, evidenced by the decreased GP values.

PAs are low-lipophilicity compounds and are most likely to interact with the polar heads of membrane lipids. In line with our results, a previous study has reported that different PAs, including CGA, decreased the lipid order of SM/Chol L_o_ domains [51]. PAs remain mostly anchored in the upper region of the membrane, with the carboxylate remaining fully solvated [51], leading to an increase in hydration and polarity at the water interface of the membranes, as indicated by the decreased GP values in our study. Additionally, their -OH and -COOH groups could alter H-bonding patterns, thereby weakening interactions between SM and Chol, inducing disordering of the L_o_ phase [51]. The integration of CGA in model membranes has been further viewed in multiple studies. Findings indicate that CGA primarily incorporates into the hydrophilic region of the membrane, altering the packing order of lipid polar head groups [52]. Its surface activity stimulates insertion of CGA into cholesterol-enriched monolayers, leading to a less-ordered phase state, resembling cholesterol-free conditions [53]. Other PAs present in the tested fractions contained various isomers of diCQA. Currently, available data are limited to molecular dynamics simulations of 3,5-diCQA interactions with lipid bilayers. These simulations reveal that 3,5-diCQA inserts more deeply into the membrane, with its quinic acid core forming hydrogen bonds at the membrane surface, while the caffeoyl aromatic rings engage in hydrophobic interactions within the lipid interior. This orientation allows 3,5-diCQA to anchor simultaneously in both polar and non-polar regions of the bilayer, potentially affecting membrane fluidity and lipid phase behavior [54]. Given the structural similarities of other isomers (e.g., 1,5-, 3,4-, and 4,5-diCQA), these molecules may exhibit comparable interactions with membranes.

In the highly fluid L_d_ phase composed of EggPC, both FGs and PAs were observed to enhance lipid order and decrease membrane fluidity, in contrast to their disordering effects on the raft-like L_o_ phase. These phase-specific effects are consistent with previous studies demonstrating that PPs can modulate membrane fluidity depending on the lipid composition and packaging. For instance, in tightly packed, saturated lipid models such as DPPC, flavonoids have been shown to increase membrane fluidity and shift the lipids’ T_m_ [48], similar to our observations. However, in membranes composed of unsaturated lipids such as SLPC, flavonoids tend to exhibit a rigidifying effect [55]. These contrasting effects are attributed to differences in the depth of compound insertion. In saturated membranes like DPPC, flavonoids predominantly associate with the polar headgroup region at the lipid–water interface [56]. In contrast, in unsaturated systems such as POPC [57] and SLPC [55], they are more likely to penetrate the hydrophobic acyl chain region, thereby altering lipid dynamics in a different manner. These contrasting behaviors highlight the complex interplay among polyphenol structure, lipid saturation, and bilayer phase in determining membrane activity.

To date, the influence of FGs and PAs on raft formation has received limited attention. Data on this matter is particularly valuable given the functional importance of lipid rafts in processes such as membrane protein clustering and signal transduction. Our findings demonstrate that both FGs and PAs significantly increase the raft-like L_o_ phase fraction in a heterogeneous membrane model composed of EggPC/EggSM/Chol. These results indicate that both PPs can enhance raft formation, but the degree of enhancement is concentration-dependent and varies with lipid composition, particularly the presence of Chol. In the low-Chol model (40/40/20), FGs and PAs showed comparable effects, suggesting that the compounds behave similarly in this lipid environment regardless of their structural differences. However, notable differences emerged in the high-Chol model (33/33/34), where PAs displayed a clear concentration-dependent enhancement of raft domain formation. This suggests a greater efficacy of PAs in segregating SM and in Chol-rich three-component systems, likely due to their polar anchoring and ability to modulate headgroup interactions at the membrane surface.

### 3.2. Antioxidant Activity of PPs Against Oxidative Damage of PUFAs

Both FGs and PAs provided a significant protective effect, reducing the percentage of oxidized lipids nearly fourfold compared to controls. At the highest concentration tested, the PPs preserved approximately 80% of the initial fluorescence, with FGs demonstrating a more pronounced protective effect. The antioxidant properties of PPs are primarily attributed to their chemical structures, particularly the presence of hydroxyl (-OH) groups [58]. These highly reactive groups can donate an electron and/or a hydrogen atom [59], thereby scavenging free radicals and neutralizing reactive oxygen species (ROS) such as hydroxyl radicals (•OH) and peroxyl radicals (LOO•). These ROS play a critical role in the initiation and propagation of lipid peroxidation, and their suppression by FGs and PAs highlights their protective potential against oxidative damage [58]. The antioxidant properties of PPs are linked to their direct interactions and incorporation into the membrane bilayer. The ability of both FGs and PAs to integrate deeper into the fluid phases of the membrane, where polyunsaturated fatty acids predominate, allows them to exert effective antioxidant activity near the site of lipid peroxidation. The engagement in interactions with the hydrophilic and hydrophobic regions of the membrane bilayer allows both PPs to reduce free radicals near the cell membrane, hinders their diffusion into the membrane interior, and terminates the chain reactions of lipid peroxidation [59]. 

### 3.3. Effects of FGs and PAs on the Lipid Order of Cell Membranes

Treatment with FGs resulted in a decrease in membrane order in both cell lines, as indicated by lower GP values. The most pronounced effect was observed in A549 cells treated with 200 µg/mL of FGs. This suggests that FGs may preferentially integrate into the membranes of cells with more fluid membranes, such as the A549 cells, where they might disrupt lipid packing. In contrast, PAs induced a concentration-dependent fluidization of A549 membranes, with lower concentrations leading to an increase in fluidity. In MDCKII cells, membrane fluidity increased at 100 µg/mL of PAs, but a slight decrease in fluidity was observed at 200 µg/mL, indicating a concentration-dependent biphasic effect. This suggests that at higher concentrations, PAs may stabilize the membrane by promoting lipid–lipid interactions, whereas at lower concentrations, they act as membrane spacers, increasing fluidity.

These findings indicate that FGs and PAs can significantly modulate membrane properties, with their effects varying depending on the initial membrane composition. FGs appear to have a stronger impact on membranes with inherently lower lipid order, as seen in the A549 cells. The ability of both FGs and PAs to modulate membrane fluidity could influence various cellular processes, such as membrane-associated signaling and the dynamics of lipid rafts. Future studies could explore these effects further, particularly in cancerous cells, to understand how FGs and PAs might be leveraged in therapeutic contexts. Additionally, examining how these compounds interact with specific membrane components could provide further insights into their potential biomedical applications.

## 4. Materials and Methods

### 4.1. Lipids

Sphingomyelin from chicken egg yolk (EggSM), № S-0756, and cholesterol № C8667-5G were purchased from Sigma-Aldrich (Sofia, Bulgaria). L-α-Phosphatidylcholine from egg yolk (EggPC) was from Avanti Polar Lipids.

### 4.2. Fluorescent Dyes

Laurdan (6-dodecanoyl-N, N-dimethyl-2-naphthylamine), DPH (1,6 Diphenyl-1,3,5-hexatrien), and TEMPO (2,2,6,6-tetramethylpiperidin-1-yl)oxyl) were bought from Sigma-Aldrich. *Cis*-parinaric acid (*cis*-PnA) [(9Z,11E,13E,15Z)-octadecatetraenoic acid] was obtained from Molecular Probes (Eugene, OR, USA).

### 4.3. Buffer

10 mM HEPES with 150 mM NaCl (pH 7.4). HEPES was supplied by Sigma-Aldrich (Sofia, Bulgaria).

### 4.4. Cell Culture Lines 

Madin-Darby Canine Kidney II (MDCKII, ATCC) cells are of non-cancerous cell line, derived from canine kidney epithelium. They form well-defined, tight junctions and have a raft-enriched plasma membrane. The cancerous A549 cells (ATCC) originate from human alveolar carcinoma. Their cytoplasm is enriched in organelles and lamellar bodies, and active cytosis is executed. Both cell lines were grown in 25 cm^2^ flasks (CELLSTAR^®^, GREINER BIO-ONE, Kremsmünster, Austria) using an incubator Sanyo MCO–18 AC under standard conditions (humidified atmosphere with 5% CO_2_, at 37 °C). The cell culture medium was Dulbecco’s Modified Eagle Medium (DMEM), supplemented with 10% fetal bovine serum (FBS) and 1% (*v*/*v*) antibiotic-antimycotic solution (penicillin—100 U/mL, streptomycin-100 μg/mL, and amphotericin B—0.25 μg/mL). Before treatment with the plant extracts, cells were plated out onto 9 cm Petri dishes.

### 4.5. Plant Extracts

#### 4.5.1. Plant Material

Wild-growing *I. oculus-christi* L. plants were collected in the flowering stage in Bulgaria and were identified by Dr. Ina Aneva (Institute of Biodiversity and Ecosystem Research, BAS, Sofia, Bulgaria). A voucher specimen (SOM 1360) was deposited in the Herbarium of the Institute of Biodiversity and Ecosystem Research, BAS, Bulgaria.

#### 4.5.2. Extraction and Fractionation of Plant Extract

Air dried and ground flowers of *I. oculus-christi* L. (180 g) were consequently extracted with chloroform (2 × 2L) and methanol (2 × 1L) at room temperature for 24 h each. The corresponding chloroform (7.4 g) and methanol (10.6 g) extracts were obtained after filtration and evaporation of the solvent under reduced pressure. A portion of the methanol extract (2.5 g) was dissolved in methanol (15 mL) and centrifuged at 5800 rpm in order to remove insoluble parts. A clear methanolic solution was concentrated up to 5 mL and applied to a Sephadex LH20 column (equilibrated with MeOH). The elution was performed with methanol and monitored by TLC (CHCl_3_:CH_3_OH:H_2_O, 60:20:4 and EtOAc:HCOOH:CH_3_COOH:H_2_O, 100:11:11:26, visualization of the spots by spraying with conc. H_2_SO_4_ or NP (Diphenylboric acid β-aminoethyl ester) reagent, heating at 105 °C and monitoring at daylight and 366 nm. Two main fractions, A and B, namely non-phenolic (1.5 g) and phenolic (0.3 g) fractions, were collected. Further, fraction B was separated by MPLC on LiChroprep RP-18 column and elution with increasing concentrations of CH_3_OH in H_2_O (20 to 80%) to give four subfractions (TLC control). Portions of the subfractions were used further for the isolation of individual compounds by prep. TLC and/or CC, as described in [60], comparison of fraction, eluted with 20% CH_3_OH in H_2_O (fraction enriched in phenolic acids (PAs)) with authentic standards (purchased from Phytolab GmbH & Co.KG, Vestenbergsgreuth, Germany) confirmed the presence of chlorogenic and 1,5-, 3,5-, 4,5-, and 3,4-dicaffeoylquinic acids. TLC comparison of the fraction, eluted with 60% CH_3_OH in H_2_O (fraction enriched in flavonoid glycosides (FGs)), with the isolated compounds (structures proven by NMR) confirmed the presence of nepetrin and hispidulin-7-O-glucoside. In this study, these two fractions were dissolved in 100 μL of dimethyl sulfoxide (DMSO) to a stock concentration of 1 mg/mL.

### 4.6. Preparation of Large Unilamellar Vesicles (LUVs) 

Lipid mixtures were prepared from stock solutions (10 mg/mL) in chloroform/methanol (1:1, *v*/*v*). Vesicles were formed from different lipid compositions: pure EggPC and EggSM, binary mixtures of EggSM/Chol (1/1), and ternary mixtures of EggPC/EggSM/Chol in varying ratios (40/40/20, 50/25/25, and 33/33/34). FGs and PAs were added to the lipid mixtures in different lipids/PPs ratios—250/1 (4 μL), 100/1 (10 μL), and 50/1 (20 μL). The organic solvents were evaporated under a nitrogen stream and further removed under a vacuum for several hours to ensure complete solvent evaporation. The dried lipid films were hydrated in HEPES buffer (10 mM HEPES, 150 mM NaCl, pH 7.4) to a final lipid concentration of 1 mM. For mixtures containing Chol and EggSM, the HEPES buffer was preheated to 60 °C to facilitate lipid miscibility. The hydration process involved heating in a 60 °C water bath for 5 min, vortexing for 1 min, sonication in an ultrasonic bath for 1 min, and cooling on ice for 5 min. These steps were repeated three times to ensure thorough lipid mixing. The resulting multilamellar vesicles (MLVs) were further processed using a LiposoFast extruder equipped with polycarbonate filters (Avestin Inc., Ottawa, Canada). The MLV dispersion was initially extruded through an 800 nm pore filter, followed by a 200 nm filter to obtain uniformly sized LUVs. The obtained LUV dispersions were used on the same day of their preparation [61]. The lipid-to-polyphenol molar ratios of 250/1, 100/1, and 50/1 were selected to approximate physiologically relevant conditions, where lipid concentrations in membranes are typically in the mM range, and polyphenols, despite low bioavailability, can reach µM concentrations in plasma following dietary intake [11,13,16]. These ratios also reflect potential local accumulations under chronic consumption. In addition, our experiments confirmed that these concentrations enable the detection of distinct biophysical effects according the sensitivity of fluorescence-based techniques.

### 4.7. DPH—Polarization Fluorescence Spectroscopy 

Depolarization of the emitted fluorescence of DPH is a reliable technique to characterize thermotropic and structural dynamics of the hydrophobic core of the model membranes. DPH is an apolar membrane dye that is embedded in the acyl chain region of the bilayer, oriented approximately perpendicular to the membrane surface. The polarization/depolarization of the emitted fluorescence is assessed by steady-state fluorescence anisotropy measurements. The fluorescence anisotropy (*r*) provides an insight into the restriction of DPH rotational motion during its excited state lifetime, which is influenced by the packing density and fluidity of the lipid acyl chains. When DPH is embedded in a rigid (ordered) lipid environment, its rotational motion is restricted. As a consequence, the fluorescence polarization is minimized, and the anisotropy is high. Conversely, when DPH is in a mobile (disordered, fluid) lipid environment, the fluorophore will undergo a rotation during its excited state lifetime, resulting in depolarization of the emitted fluorescence and a decrease in the fluorescence anisotropy [62,63,64].

DPH was dissolved in tetrahydrofuran and was added to the lipid mixtures upon LUVs preparation, so the lipids/DPH ratio was 200/1. The measurements were carried out in a quartz cuvette, where the labeled LUV suspension was diluted to 0.5 mM in HEPES buffer. DPH fluorescence anisotropy in EggSM LUVs and EggSM/Chol LUVs with different lipids/FGs and lipids/PAs ratios was measured within the temperature range from 20 to 51 °C. The first four measurements were taken at 3 °C increments (20–29 °C). Thereafter, the temperature was increased by 2 °C before each subsequent measurement (29–51 °C). The excitation of monochromatic light was generated by a polarizer whose polarizing axis was oriented vertically to the light path. The excitation wavelength for DPH was set at 358 nm, and the emission was measured at 430 nm. The emission intensities were detected via an analyzer with polarization axes oriented parallel and perpendicular to the polarized excitation light. The emission was detected at a right angle to the excitation beam. The fluorescence anisotropy was calculated using the intensities of the polarized emission as follows [63,65]:

*r* = (*I_vv_* − *GI_vh_*)/(*I_vv_* + 2*GI_vh_*) (1)

where *v* and *h* stand for vertical and horizontal one. *v* denotes the polarization of the excitation light, whereas *h* denotes the polarization of the emission signal corresponding to the orientation of the excitation and the emission polarizers. The grating factor *G* is *I_hv_*/*I_hh_* and accounts for the sensitivity of the instrument towards the vertically and horizontally polarized light. DPH anisotropy values range from −0.2 (low anisotropy) to + 0.4 (high anisotropy) [62].

### 4.8. DPH Quenching by TEMPO

DPH fluorescence quenching by TEMPO was employed to assess the effect of FGs and PAs on the formation of nano-scaled L_o_ raft phases in the heterogeneous membranes, composed of EggPC/EggSM/Chol. DPH exhibits no preference between the lipid phases in heterogeneous membranes; therefore, it is evenly distributed in the two co-existing phases in the bilayer [66]. On the other hand, TEMPO is water-soluble and diffuses into the polar and fluid L_d_ phase. Consequently, TEMPO quenches the fluorescence of DPH in the L_d_ phase. The remaining DPH fluorescence after quenching is proportional to the fraction of the ordered domains (*Q*) and was calculated as follows:

*Q* = *F*/*F*_0_ (2)

where *F* and *F_0_* are the fluorescence intensities of DPH in the presence and absence of TEMPO [67], respectively. The final TEMPO concentration in samples was 2 mM.

The quenching of DPH fluorescence by TEMPO was measured using a Synergy^TM^2 (BioTek Instruments, Vermont, USA) spectrofluorometer in a 96-well plate (Greiner, CELLSTAR^®^, Merck KGaA, Darmstadt, Germany) at 37 °C. The lipids/DPH ratio was 1000/1. Fluorescence was recorded at 360/40 nm excitation and 460/40 nm emission. Samples were run in triplicate.

### 4.9. Laurdan Fluorescence Spectroscopy 

Laurdan fluorescence was used to investigate lipid order changes in both model and cell membranes. Laurdan is a polarity-sensitive dye whose functionality is based on solvent dipole relaxation. Its fluorescent naphthalene moiety possesses a dipole moment, which is localized at the hydrophilic–hydrophobic interface of the membrane bilayer. Upon excitation, Laurdan’s dipole moment increases, inducing the reorientation of solvent dipoles around the alcohol backbone of membrane lipids. Changes in membrane phase states alter the concentration and molecular dynamics of these solvent dipoles, thereby affecting Laurdan’s emission. In disordered lipid environments, Laurdan exhibits a red-shifted emission centered at 490 nm, whereas in ordered membranes, its emission is blue-shifted and centered at 440 nm [68,69,70].

The Laurdan emission maximum exhibits a 50 nm shift and is used to calculate the generalized polarization (*GP*) as follows [70]:

*GP* = (*I*_440_ − *I*_490_)/(*I*_440_
*+ I*_490_) (3)

where *I*_440_ and *I*_490_ represent the fluorescence intensities measured at the maximum emission wavelengths of Laurdan in ordered phases (440 nm) and the disordered phases (490 nm). High and positive GP values (+1) are indicative of ordered lipid bilayers, whereas low and negative values report bilayers in a fluid phase state. Experimentally, GP values range from 0.6 to −0.2 [69].

The steady-state fluorescence was measured using a Hitachi Fluorescence Spectrophotometer F-7000 (Hitachi High-Technologies Corporation, Tokyo, Japan), equipped with a xenon lamp with excitation and emission slits set to 5 nm. The temperature in a cuvette containing a magnetic stir bar was measured by using a platinum thermometer and maintained under the control of a water-circulating bath. The measurements were performed with quartz cuvettes. The excitation wavelength of Laurdan was set at 355 nm, and the emission spectra were recorded from 390 to 600 nm.

Laurdan fluorescence spectroscopy for model membranes: A stock solution of Laurdan of 707 μM was prepared in chloroform/methanol (9:1 *v*/*v*). Laurdan solution was then added to the lipid mixtures in the organic solvents upon vesicle formation at a lipids/Laurdan ratio of 200/1. The labeled vesicle dispersion was diluted to a concentration of 0.2 mM. The measurements were performed within the temperature range from 20 to 50 °C with a gradual increase of 5 °C for each measurement.

Laurdan fluorescence spectroscopy for cell membranes: The membranes were labeled according to an optimized protocol from Parasassi et al., 1993 [70]. Cells with a concentration of 3 × 10^6^ were washed two times by centrifugation at 1500 rpm for 5 min in 2.5 mL PBS and then resuspended in 2.5 mL of PBS. Then, 0.5 µL of a 0.25 mM solution of Laurdan in DMSO was added to the cell suspensions, so the final concentration of Laurdan and DMSO was 0.05 µM and 0.02%, respectively. The samples were incubated for an hour in the dark with mild stirring. After the incubation time, the samples were centrifuged again, the supernatant was removed, and 2.5 mL PBS was added. The measurement was performed in 1 mL quartz cuvettes at a physiological temperature of 37 °C.

### 4.10. Cis-Parinaric Acid Fluorescence Assay 

This was performed as described before [71].

### 4.11. Statistical Analysis 

This was performed using OriginPro 9.0. The mean values of each measured parameter at 37 °C were compared using Pair-Sample *t*-Test. Statistically significant differences based on the Tukey test linked to the control group and between the two investigated PPs (FGs and PAs) at each lipids/PPs ratio were determined at *p* < 0.05 (*), and no significant differences were marked as ns.

## 5. Conclusions

FGs and PAs alter the phase transition of SM, a key phase-regulatory lipid in membranes. FGs disrupt lipid packing, increasing bilayer fluidity, while PAs enhance it at the acyl chain level. Both compounds influence phase transitions by modulating membrane heterogeneity, confirming their partitioning into the bilayer. In the raft-like L_o_ phase, they act as spacers, increasing fluidity and polarity while weakening lipid–lipid interactions. Conversely, in the highly fluid L_d_ phase, they promote tighter lipid packing and increased rigidity. This dual effect is driven by their ability to penetrate the bilayer at different depths, thereby altering membrane structural dynamics.

The findings further demonstrate that both FGs and PAs promote the formation of raft-like L_o_ domains in heterogeneous membrane models, suggesting their potential role in modulating membrane structure and raft-associated processes. The extent of raft enhancement was concentration-dependent and varied based on lipid composition, particularly cholesterol content. In the low-Chol model, both compounds exhibited similar effects; however, in the high-Chol model, PAs exhibited a direct concentration-dependent effect, suggesting a stronger ability to enhance raft formation in Chol-rich membranes.

Both FGs and PAs exhibited strong antioxidant activity, effectively protecting unsaturated lipids from oxidative damage by inhibiting lipid peroxidation. FGs demonstrated a more pronounced protective effect, preserving lipid integrity to a greater extent. By limiting oxidative stress and preventing lipid peroxidation, these PPs play a crucial role in maintaining membrane stability and protecting lipids from oxidative damage, highlighting their potential therapeutic and biomedical applications.

FGs decreased membrane lipid order in both cell lines, with the most pronounced effect observed in the highly fluid membranes of A549 cells. This suggests that FGs integrate into the bilayer and disrupt lipid packing, enhancing membrane polarity. In contrast, PAs had a stronger impact on the more fluid A549 membranes but exhibited a biphasic effect in the more ordered MDCKII membranes. These findings highlight the ability of FGs and PAs to remodel the membrane structure and organization differently depending on lipid composition, with consequences for the lipid raft architecture, and associated signaling pathways.

## Figures and Tables

**Table 1:** Main transition temperature (T_m_) and Lorentzian peak width of control EggSM LUVs and PP-containing ones at different ratios (fitting parameters in SI). Results are presented as means ± standard deviations (SD) of three independent measurements per sample (*n* = 3). * denotes a statistically significant difference (*p* < 0.05) between the control and the PP-containing sample.

Sample	T_m_	Lorentzian Peak Width
EggSM (control)	39.3 ± 0.6	4.4 ± 0.5
EggSM/FGs		
250/1	38.4 ± 0.7 *	6.5 ± 0.7 *
100/1	39.5 ± 0.8	5.2 ± 0.4 *
50/1	38.2 ± 0.4 *	2.3 ± 0.5 *
EggSM/PAs		
250/1	39.9 ± 0.6	4.5 ± 0.8
100/1	41.2 ± 0.9 *	6.5 ± 0.9 *
50/1	41.3 ± 0.7 *	3.3 ± 0.6 *

## Data Availability

Data are contained within the article.

## Supplementary Materials

The following are available online at https://www.mdpi.com/article/10.3390/molecules30132740/s1, Table S1: Sigmoid fit parameters describing the temperature dependence of DPH anisotropy during the L_β_/L_α_ phase transition of EggSM membranes, without and with polyphenols (FGs (Figure 1) and PAs (Figure 3)), measured over the temperature range of 20–50 °C, Table S2: Lorentz fit parameters for the first derivative of DPH anisotropy during the L_β_/Lα phase transition in EggSM LUVs without and with polyphenols (FGs (Figure 2) and PAs (Figure 4)); Table S3: Linear fit parameters for the temperature dependence of DPH anisotropy of raft-like L_o_ phase without and with PPs (FGs and Pas) measured across a temperature range of 20–50 °C.

## Abbreviations

The following abbreviations are used in this manuscript:

DMSO	Dimethyl sulfoxide
FBS	Fetal bovine serum
LUV	Large unilamellar vesicles
DPH	6-diphenyl-1,3,5-hexatriene
PPs	Polyphenols
FGs	Flavonoid glycosides
PAs	Phenolic acids
Chol	Cholesterol
EggSM	Sphingomyelin from chicken egg yolk
EggPC	L-α-Phosphatidylcholine from egg yolk
