# Supplementary material for: Flavonoid Glycosides and Phenolic Acids from Inula Oculus-Christi Modulate Membrane Organization and Provide Antioxidant Protection
Source: Molecules. 2025 Jun 25;30(13):2740. doi: 10.3390/molecules30132740 (PMC12250669; doi:10.3390/molecules30132740)
Supplement: Supplementary file 1 [file molecules-30-02740-s001.zip › molecules-3660555-supplementary.pdf]

Supplementary material

1. Effects of PPs on the Phase Transition of EggSM

1.1 Sigmoid Fit of the Experimental Data

To quantify the phase transition temperature, the experimental data on the temperature-dependent DPH anisotropy of EggSM membranes, with and without polyphenols (Figures 1 and 3), were fitted using a Boltzmann sigmoid function. The equation used for the fitting is provided in the legend of Table S1, which summarizes the corresponding fit parameters. This approach allows accurate determination of the transition midpoint (x_0) and cooperativity (dx) for each sample.

Table S1. Sigmoid fit parameters describing the temperature dependence of DPH anisotropy during the L_β/L_α phase transition of EggSM membranes, without and with polyphenols (FGs (Figure 1) and PAs (Figure 3)), measured over the temperature range of 20–50 °C.

Fitting equation: $y=A_2+(A_1-A_2)/(1+exp((x-x_0)/dx))$

Sample	$A_1 \pm$ SE	$A_2 \pm$ SE	$x_0 \pm$ SE (°C)	dx ± SE	Adjusted R^2
EggSM Control	0.29 ± 0.00	0.12 ± 0.01	39.08 ± 0.66	2.21 ± 0.6	0.96
EggSM/FGs (250:1)	0.27 ± 0.00	0.09 ± 0.00	38.22 ± 0.25	2.03 ± 0.22	0.99
EggSM/FGs (100:1)	0.28 ± 0.00	0.10 ± 0.00	38.17 ± 0.32	1.33 ± 0.27	0.98
EggSM/FGs (50:1)	0.29 ± 0.01	0.08 ± 0.02	38.15 ± 0.69	3.39 ± 0.79	0.97
EggSM/PAs (250:1)	0.30 ± 0.01	0.11 ± 0.01	40.14 ± 0.56	1.98 ± 0.49	0.97
EggSM/PAs (100:1)	0.30 ± 0.01	0.12 ± 0.02	40.48 ± 0.76	2.31 ± 0.67	0.96
EggSM/PAs (50:1)	0.27 ± 0.01	0.14 ± 0.01	40.53 ± 0.73	1.98 ± 0.62	0.95

A_1 and A_2 represent the upper and lower asymptotes of the fit, respectively; x_0 is the temperature at the midpoint of the transition; dx reflects the slope of the transition curve; SE: Standard Error from the non-linear curve fitting.

1.2. Fit of the First Derivative of Sigmoid Curve

The first derivative of the sigmoid curve provides a more precise estimation of the transition width, as it directly reflects the rate of change in membrane order. Unlike the original sigmoid fit, which describes the cumulative transition, the derivative highlights the sharpness and cooperativity of the phase transition. Fitting this derivative with a Lorentz function allows more accurate determination of the transition width. Table S2 describes the parameters of the fit. While both fits accurately reflect the changes in transition midpoint, the first derivative of the sigmoid curve provides a more precise estimation of the transition width.

Table S2. Lorentz fit parameters for the first derivative of DPH anisotropy during the L_β/Lα phase transition in EggSM LUVs without and with polyphenols (FGs (Figure 2) and PAs (Figure 4)).

Fitting equation: $y = y_0 + (2A/\pi)(\omega/(4(x-xc)^2 + \omega^2))$

Sample	x_c ± SE (°C)	ω ± SE	Adjusted R^2
EggSM Control	39.31 ± 0.58	4.39 ± 0.47	0.88
EggSM/FGs (250:1)	38.37 ± 0.65	6.47 ± 0.66	0.86
EggSM/FGs (100:1)	39.45 ± 0.77	5.18 ± 0.39	0.82
EggSM/FGs (50:1)	38.20 ± 0.36	2.27 ± 0.45	0.79
EggSM/PAs (250:1)	39.92 ± 0.60	4.51 ± 0.78	0.82
EggSM/PAs (100:1)	41.21 ± 0.91	6.52 ± 0.91	0.74
EggSM/PAs (50:1)	41.33 ± 0.74	3.32 ± 0.62	0.75

x_c represents the center of the transition (peak temperature); w is the width of the peak (related to cooperativity); SE = Standard Error from nonlinear fitting.

2. **Effects of FGs and PAs on the Lipid Order of the L_o Raft-like Phase, Composed of EggSM/Chol**

A linear regression analysis was performed to examine the temperature dependence of DPH anisotropy in EggSM/Chol (1:1 molar ratio) membranes without and with PPs (Fig. 5 and 6 from the manuscript).

Table S3. Linear fit parameters for the temperature dependence of DPH anisotropy of raft-like L_o phase without and with PPs (FGs and Pas) measured across a temperature range of 20–50°C.

Fitting equation: $y = a + b \cdot x$, where y is DPH anisotropy and x is temperature (°C).

Sample	a (Intercept ± SE)	b (Slope ± SE)	Adjusted R^2
EggSM/Chol (Control)	0.321 ± 0.016	$-0.001 \pm 4.4 \times 10^{-4}$	0.325
Lipids/FGs (250/1)	0.302 ± 0.019	$-0.001 \pm 5.5 \times 10^{-4}$	0.198
Lipids/FGs (100/1)	0.284 ± 0.012	$-1.0 \times 10^{-4} \pm 3.4 \times 10^{-4}$	–0.101
Lipids/FGs (50/1)	0.281 ± 0.010	$-4.2 \times 10^{-5} \pm 2.9 \times 10^{-4}$	–0.109
Lipids/PAs (250/1)	0.271 ± 0.014	$-0.001 \pm 3.8 \times 10^{-4}$	0.306
Lipids/PAs (100/1)	0.260 ± 0.010	$-1.9 \times 10^{-4} \pm 3.3 \times 10^{-4}$	–0.045
Lipids/PAs (50/1)	0.252 ± 0.012	$-4.8 \times 10^{-4} \pm 4.1 \times 10^{-4}$	0.102
